# Comparisons of Different Multimorbidity Measures to Predict Physical Function Among Middle-Aged and Older Chinese

**DOI:** 10.1093/geroni/igab046.2082

**Published:** 2021-12-17

**Authors:** Hui-wen Xu, Yan Luo, Zi-Ting Huang, zi-shuo Chen, He-Xuan Su, Beibei Xu

**Affiliations:** 1 Peking University School of Public Health, Beijing, Beijing, China (People's Republic); 2 Peking university School of Public Health, Beijing, Beijing, China (People's Republic); 3 Peking University Medical Informatics Center, Beijing, Beijing, China (People's Republic)

## Abstract

Multimorbidity has been associated with declined physical function. Several methods have been used to measure multimorbidity. However, few studies have compared the associations between different multimorbidity measures and physical function. We aimed to examine and compare the associations between different multimorbidity measures and physical function. We included 16,117 participants aged ≥45 from China Health and Retirement Longitudinal Survey (CHARLS) 2011-2018. Multimorbidity was defined as the co-existence of two or more of fifteen chronic conditions in an individual and measured by condition count at penultimate, multimorbidity patterns at penultimate (examined by exploratory factor analysis) and multimorbidity trajectories from baseline to penultimate living interview (examined by the group-based trajectory model). Physical function was assessed by activities of daily living or instrumental activities of daily living at the last follow-up. Logistic regression models were conducted for establishing prediction models in the training set. We used c-statistic, Integrated Discrimination Improvements (IDI) and Net Reclassification Index (NRI) to compare the performance of different models in the testing set. After adjusting for age and gender, compared with those without any conditions, participants with multimorbidity measured by three methods all had higher risks of poor physical function in the training set. Compared with the model using condition count (c-statistic=0.749), models using multimorbidity trajectory (c-statistic=0.712, IDI=-0.03, NRI=-0.31) and the multimorbidity pattern (c-statistic=0.739, IDI=-0.01, NRI=-0.16) showed poor predictive power (all p<.05). In our study, condition count has the best predictive performance for poor physical function over short time period. It is a simple and useful tool to assess multimorbidity.

